# Does robotic intersphincteric resection better preserve anal function in low rectal cancer: a systematic review and meta-analysis?

**DOI:** 10.3389/fmed.2026.1778921

**Published:** 2026-05-21

**Authors:** Wenjie Zhou, Xueting Wang, Jie Dan, Mingjie Zhu, Qian Liao, Ming Li, Ke Liu, Yonghong Wang

**Affiliations:** 1 Department of Gastrointestinal Surgery, The People’s Hospital of Leshan, Leshan, China; 2 Labor Union Office, The People’s Hospital of Leshan, Leshan, China

**Keywords:** functional outcomes, intersphincter resection, meta-analysis, rectal cancer, robotic

## Abstract

**Backgroud:**

Intersphincteric resection (ISR) is an important and challenging sphincter-preserving procedure for patients with low rectal cancer. Preserving anal function is more important than preserving its appearance. Owing to its unique advantages, the robotic platform makes ISR safer and less invasive. Whether this advantage is reflected in functional aspects is worth further exploration.

**Methods:**

PubMed, Cochrane Library and Embase were systematic researched for articles published relevant literature. The literature was screened independently by two groups, and data on functional, perioperative, and oncology outcomes were extracted and evaluated for bias. Meta-analysis was performed using Revman5.4 software.

**Results:**

Ten studies with a total of 1279 patients were included in our meta-analysis, and Funnel plots suggested no major publication bias. The robotic ISR group had lower Wexner scores [MD = –1.46, 95%CI (–2.03 ∼–0.88), *p* < 0.001] than the laparoscopic ISR group, but with no significant difference compared to other functional outcomes. The robotic ISR group performed better in preserving the integrity of the rectal mesentery [OR = 3.08, 95%CI (1.72, 5.53), p = 0.0002], had less intraoperative bleeding [MD = –11.47, 95%CI (–17.25, –5.68), *p* = 0.0001], and had fewer postoperative complications [OR = 0.71, 95%CI (0.53, 0.94), *p* = 0.02], although the operation took longer [MD = 0.76, 95%CI (0.62∼0.91), *p* < 0.001]. No significant differences were observed in the long-term oncological outcomes between the two groups.

**Conclusion:**

Robotic ISR has potential advantages in preserving the anal function, with fewer complications and similar oncological outcomes compared with Laparoscopic ISR. Due to limitations in sample size and study design, the results of this study still require validation through randomized controlled trials.

**Systematic review registration:**

https://www.crd.york.ac.uk/PROSPERO, identifier CRD420251012991.

## 1 Introduction

Colorectal cancer is one of the most common malignancies, with the third and second highest morbidity and mortality rates worldwide, respectively, in 2022 (1) and rectal cancer cases account for roughly half of all cases in China. A cross-sectional study revealed that curing the tumor and not having a permanent stoma were the top concerns of patients (2). Intersphincteric resection (ISR) is an important sphincter-preserving procedure for patients with low rectal cancer. The main indication is low rectal cancer when the tumor does not extend beyond the internal anal sphincter. ISR can be classified into three types: partial, subtotal, and total, according to the amount of internal sphincter removed (3). Decades of research have shown that the long-term oncological outcomes and safety of the ISR procedure are comparable to those of abdominoperineal resection (4, 5).

Currently, the decrease in anal function and incontinence remains an unsolved problem for ISR (6). One of these is that the narrow pelvic cavity restricts the precise execution of surgery. The external sphincter and pelvic nerves are easily damaged using laparoscopic method. Over the past decade, robotic surgeries have been carried out in some centers. Robotic platforms may facilitate ISR in low rectal cancers because of their more flexible robotic arms and better field of view. Robot platforms allow the surgeon to sit which can prevent surgeons from becoming fatigued, showing an independent association with a higher ISR incidence comparable to that of laparoscopy (7). However, whether these advantages are reflected in anal function, oncology, and complications remains unclear. At the same time, robotic surgery also has some limitations. It is costly, lack tactile feedback, and require a long setup time before the operation. Accordingly, this study collated the most recent literature pertaining to systematic reviews and meta-analyses, as detailed below.

## 2 Methods

This study was conducted according to the current preferred reporting items for systematic reviews and meta-analyses (PRISMA) (8) and methodological quality guidelines for systematic reviews (AMSTAR) (9), and has been registered in PROSPERO(CRD420251012991). We declare that no artificial intelligence was used in the research and manuscript writing process (10).

### 2.1 Search strategy

PubMed, Cochrane Library, and Embase were systematically searched for articles published independently by two researchers. Only articles in English were retained, and human studies were identified up to April 30, 2025. Our search also included all references of all articles researched, which were retrieved in their full text. The retrieval scheme adopted the method of combining medical subject words with free words to identify relevant literature. The medical subjects were “Robotic Surgical Procedures” and “Rectal Neoplasms.” Free words were generated based on the subject terms in the MESH database of the National Library of Medicine. Other free words included “robotic,” “robot,” “da Vinci,” “Intersphincteric resection,” “ISR,” and “sphincterectomy” due to the absence of appropriate medical subject headings or the insufficiency of free words in the MeSH database. The detailed search strategy can be found in Supplementary Appendix 1. The search terms were limited to the titles and abstracts. Two researchers independently screened the retrieved literature and assessed the eligibility of each study included in the meta-analysis. Differences should be resolved through consensus and, if necessary, through meetings with the study group.

### 2.2 Inclusion and exclusion criteria

The inclusion criteria were as follows: (1) patients with a histological diagnosis of low rectal cancer; (2) all original studies using Robotic ISR as the experimental group, either prospective or retrospective; (3) Laparoscopic ISR as the treatment of choice in the laparoscopic group; (4) outcome indicators reported at least one of the following results: functional outcomes, perioperative outcomes, and oncological outcomes; and (4) the publication language was English only. (5) Newcastle-Ottawa Scale (NOS) score more than five for Cohort studies. (6) The most recent study was selected for inclusion when duplicate or overlapping articles were published by the same institution and researcher.

The exclusion criteria were as follows: (1) single-arm and other noncontrolled studies. (2) Studies with incomplete data or those unable to extract data. (3). stromal tumors, or unclear pathological types.

### 2.3 Data extraction and processing

Two researchers independently extracted the literature data. When there were differences, they are verified by a third one and the final analysis data were discussed and determined. The data extracted were as follows: (1) general data of the literature, including first researcher name, publication year, country or region, study type, propensity score, multi-center clinical study, case enrollment time, and sample size; (2) basic information of the patient, including age, body mass index (BMI), gender, lesion site, tumor node metastasis classification (TNM), American College of Anesthesiologists score (ASA), distance of the tumor from the anus(DAV), preoperativetive carcinoembryonic antigen (pre-CEA); (3) details of the operation, including operation time, blood loss, type of ISR, type of anastomotic, temporary ileostomy, conversion to open or APR.; and (5) perioperative and pathological data, including the time to flatus and stool, length of hospital stay, postoperative complications, number of lymph nodes obtained, and distance of the distal resection margin. (6) recurrence and survival analysis results, including disease-free survival (DFS), overall survival (OS), and local recurrence-free survival (LRFS). (7) functional outcomes, including anal, bladder, and sexual function. If multiple functional evaluations are available, we will conduct a meta-analysis based on the most recent one. The primary outcome of this study was functional outcome. Secondary outcomes included postoperative recovery and oncological outcomes. For continuous data with quartile or median and extreme values, the mean and standard deviation were extracted according to the methods of D. Luo, J. Shi and X. Wan, and valid data that could not be extracted were not included in the meta-analysis (11–13). If only K-M plots were provided without HR data, the HR was converted using the method of Jayne F Tierney (14). If propensity score matching (PSM) or inverse probability weighting analysis (IPTW) was performed, we only used the data after matching or weighting. For the same type of data published by the same team, we adopted data from the most recent literature.

### 2.4 Quality and risk of bias assessment

Two researchers independently evaluated the quality of the literature, and all disagreements were resolved by consensus agreement. For cohort studies, the Newcastle-Ottawa Quality Assessment Tool (NOS scale) was used for quality and risk of bias assessment, which rated the quality of eight items in three domains: selectivity (up to 4 points), comparability (up to 2 points), and outcome (up to 3 points). The higher the score, the higher the quality.

### 2.5 Statistical analysis

Statistical analysis was conducted using Revman 5.4 version software, provided by the Cochrane Library. Odds ratios (ORs), mean differences (MD), hazard ratios (HR), and their corresponding 95% confidence intervals (95%CI) were calculated for counting and measurement data, respectively. The Q and I^2^ tests were used to examine heterogeneity. If heterogeneity was high (*I*^2^ > 50%), pooled estimates were calculated using the random-effects method. Otherwise, a fixed random effects model is used. For cases with high heterogeneity, one-way sensitivity and subgroup analyses were used to explore the sources of heterogeneity. To test the stability of the meta-analyses, subgroup analyses were performed according to center (multicenter vs. single center), sample size (more than median vs. less than median), study type (prospective vs. retrospective), and time to publication (earlier than median time vs. later than median time). ISR type (partial vs. subtotal vs. total), country (Korea vs. other countries), and tumor distance from the anus verge (DAV < 3 cm vs. > 3 cm). A funnel plot was used to test for publication bias in more than eight studies. A *p*-value < 0.05 for the pooled data was considered significant.

## 3 Results

### 3.1 Study characteristics

**FIGURE 1:**
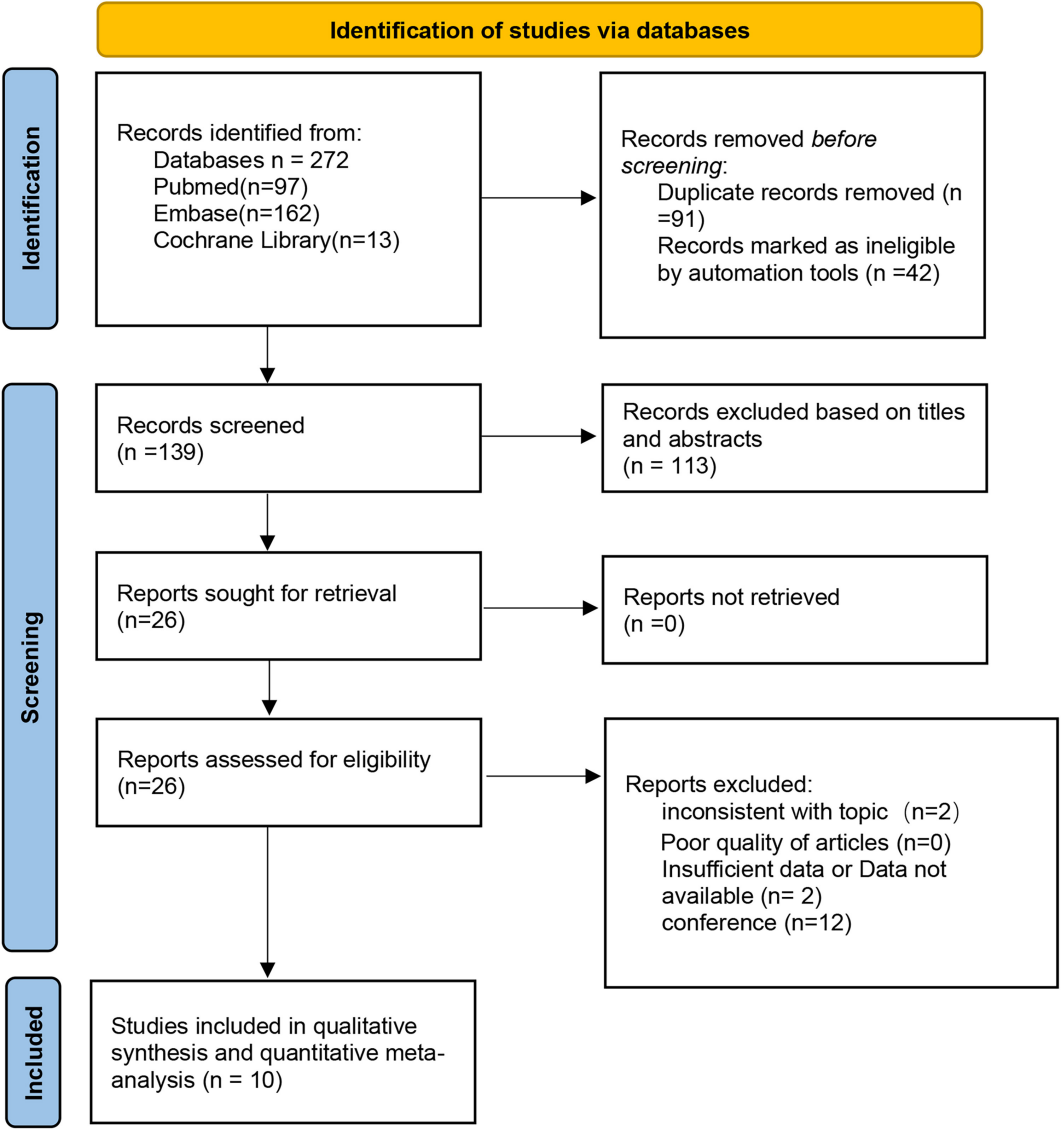
The PRISMA diagram for the selection of the studies.

A total of 272 articles were retrieved according to the initial search strategy, and ten studies were included in the meta-analysis, with 1279 cases (607 and 672 cases in the robotic and laparoscopic groups, respectively) meeting the inclusion criteria. The literature screening process is shown in Figure 1. Eight retrospective studies, one prospective study, and one cross-sectional study were included. PSM was used in two studies (15, 16), and IPTW was used in one (17). Three were multicenter clinical studies, and the others were single-center studies. Only three studies clearly demonstrated that preoperative anal function was normal or that there was no statistically significant difference between the groups. Eight studies reported on functional outcomes. The research characteristics are presented in Table 1.

**TABLE 1:** Characteristics of the studies.

Author	Year	Study type	Region	Center	NOS	Enrollment time	Sample size	Functional assessment	Baseline of anus function	Inclusion criteria characteristics
Bo et al. (18)	2025	Retrospect	China	1	8	January 2016 to July 2019	300	Bowel movement frequency, Wexner scores, LARS Scores, IPSS scores	Normal preoperative anal function	1. Differentiated adenocarcinoma; 2. Location ≤ 6 cm from the anus verge; 3. Normal preoperative anal function.
Ge et al. (22)	2025	Retrospect	China	1	6	August 2018 to August 2021	28	Kelly score	NA.	1. 18–75 years; 2. 2–5 cm from the anus verge; 3. Without history of rectal or anal surgery; 4. Without acute intestinal obstruction or intestinal perforation. 5. Well-differentiated adenocarcinoma
Sun et al. (15)	2024	Retrospect	China	2	9	January 2017 to December 2018 for Rob January 2014 to December 2018 for Lap	184	Wexner score, Kirwan’s score, major LARS	Normal preoperative anal sphincter function	1. Well-differentiated adenocarcinoma;2. Tumor located ≤ 5 cm; 3. cT1-2N0M0; 4. Normal preoperative anal sphincter function 5. Did not invasion of the dentate line
Kazi et al. (17)	2023	Cross sectional	India	1	8	July 2013 to October 2021	132	Major LARS, major Wexner, Kirwan grades	NA.	1. Non-metastatic; 2. Prophylactic diverting ostomy; 3. Stomas reversed at least six months prior to the functional assessment; 4. Location ≤ 5 cm from the anus verge.
Aliyev et al. (23)	2022	Retrospect	Turkey	multi	8	January 2011 to January 2020	115	Wexner score, Kirwan grades, Stool frequency	Absence of preoperative anal function assessment	1. Within 5 cm from the AV; 2. Adenocarcinoma; 3.18 to 75 years; 4. Synchronous tumors or distant metastasis; 5. Without preoperative fecal incontinence
Shin et al. (24)	2015	Retrospect	Korea	1	6	January 2011 to December 2014	94	NA.	NA.	NA.
Park et al. (16)	2015	Retrospect	Korea	7	9	January 2008 to May 2011	278	Bowel movements, pads and antidiarrhoeal medication, sexual dysfunction	NA.	1. Adenocarcinomas, 2. Tumor located within 4 cm of the anal verge.
Yoo et al. (19)	2015	Retrospect	Korea	1	8	September 2006 to August 2011	70	Day- and night-time leakage, urgency, frequency, and Wexner scores	NA.	Within 5 cm from the AV
Kuo et al. (21)	2014	Retrospect	Taiwan	1	6	November 2009 to July 2013	64	NA.	NA.	Received ISR
Park et al. (20)	2013	Retrospect	Korea	1	7	March 2008 to July 2011	80	Wexner score IPSS IIEF-5	Without different between groups	Curative intent during the study period

NOS, Newcastle-Ottawa Scale; FIS, fecal incontinence score; IIEF-5, international index of erectile function; IPSS, international prostate symptom score; LARS, rectal anterior resection syndrome; AV, anus verge; ISR, intersphincteric resection.

Most of the basic demographic characteristics between the two groups (including age, gender, BMI, ASA, and pre-CEA) showed no differences, as described in each study. In three studies (15, 18, 19), the tumors in the robotic ISR group were closer to the anus, and in Yang Bo’s study (18), the mean difference in DAV between the two groups was the greatest. Only one study included patients whose initial stage was stage I (15). In other studies, patients with stage T3-4 were also included, but they received neoadjuvant therapy to reduce the stage. There were still slight differences in the baseline data of a few studies between these two groups. In some robotic surgical research groups, patients had a higher BMI (19), were younger (20), had a higher proportion of preoperative neoadjuvant therapy (19–21). The characteristics of the included population are presented in Table 2.

**TABLE 2:** Characteristics of the populations in each study.

Author	Group	Sample size (N)	Male/ Female	Age (years)	BMI (kg/m2)	ASA (I/II/ ≥ III,N)	cTNM(I/II/ III,N)	Pre-CEA ( > abnormal, N, %)	DAV, (cm)	ISR type (partial/subtotal/ total, N)	nCRT (%)
Bo et al. (18)	Robotic	150	94/56	58.3 ± 10.4	23.6 ± 3.2	33/87/30	112/9/29	25(16.7)	3.94 ± 0.48	36/88/33	33(22%)
	Laparoscopic	150	96/54	60.2 ± 10.8	23.3 ± 3.7	24/95/31	95/16/39	29(19.3)	5.66 ± 0.47	40/88/22	48(32%)
Ge et al. (22)	Robotic	16	8/8	57.1 ± 15.2	23.8 ± 2.8	NA.	13/0/3	NA.	3.2 ± 1.0	13/3/0	0(0%)
	Laparoscopic	12	7/5	58.0 ± 9.5	24.5 ± 3.1	NA.	7/0/5	NA.	2.5 ± 0.9	10/2/0	1(8.3%)
Sun et al. (15)	Robotic	68	48/20	62.5 ± 8.7	23.5 (22.1∼26.3)	24/40/4	stage I only	NA.	2.9 ± 0.61	NA.	0
	Laparoscopic	68	46/22	63.1 ± 8.0	23.6 (22.7∼25.5)	16/46/6	stage I only	NA.	3.2 ± 0.69	NA.	0
Kazi et al. (17)	Robotic	130	96/34	46.42 (12.19)	23.69 (3.62)	NA.	NA.	NA.	3.3 (1.52)	partial or subtotal ISR	110(84.6%)
	Laparoscopic	132	98/34	46.34 (12.55)	23.67 (3.36)	NA.	NA.	NA.	3.3 (1.49)	partial or subtotal ISR	112(84.8)
Aliyev et al. (23)	Robotic	60	42/18	60.0 ± 10.2	23.0 ± 2.8	25/31/4	1/21/38	NA.	3.0 ± 0.6	partial ISR	60(100%)
	Laparoscopic	55	36/19	58.0 ± 9.7	24.0 ± 2.4	21/27/7	2/17/36	NA.	3.5 ± 0.7	partial ISR	52(94.5%)
Shin et al. (24)	Robotic	34	22/12	55 ± 12.8	23.7 ± 3.1	19/41/0	0/12/4/18/0 (0/I/II/III/IV)	4.0 ± 8.7	2.7 ± 0.9	NA.	23(67.6%)
	Laparoscopic	60	35/25	58 ± 10.3	23.1 ± 4.3	13/20/1	1/21/5/28/5 (0/I/II/III/IV)	2.9 ± 3.8	2.5 ± 0.7	NA.	40(66.7%)
Park et al. (16)	Robotic	106	75/31	59.6(10.8)	24.3(2.8)	48/52/6	NA.	4.3(7.7)	3.2(1.0)	NA.	68(64.2%)
	Laparoscopic	106	71/35	61.7(9.6)	23.8(3.3)	42/50/14	NA.	4.0(4.7)	3.3(1.1)	NA.	60(56.6%)
Yoo et al. (19)	Robotic	44	35/9	59.77 ± 12.33	24.13 ± 3.33	26/17/1	NA.	NA.	3.24 ± 0.78	NA.	24(54.5%)
	Laparoscopic	26	19/7	60.5 ± 10.75	21.42 ± 3.13	15/11/0	NA.	NA.	3.71 ± 0.89	NA.	7(26.9%)
Kuo et al. (21)	Robotic	36	15/21	55.9 (30–89)	NA.	0/33/3	NA.	NA.	3.83 (1.5–5.0)	NA.	28(77.8%)
	Laparoscopic	28	11/17	54.9 (25–88)	NA.	4/22/2	NA.	NA.	3.71 (2.0–6.0)	NA.	28(100%)
Park et al. (20)	Robotic	40	28/12	57.3 (12.1)	23.9 (2.4)	27/9/4	NA.	4.2 (7.9)	3.4 (1.1)	NA.	32(80%)
	Laparoscopic	40	25/15	63.6 (10.6)	24.3 (3.1)	24/14/2	NA.	3.3 (3.5)	3.6 (1.3)	NA.	20(50%)

BMI, body mass index; ASA, American society of anesthesiologists; DAV, Tumor distance from anus verge; Pre-CEA, preoperative carcino-embryonic antigen; ISR, intersphincteric resection; nCRT, neoadjuvant chemoradiotherapy NA, Not available.

### 3.2 Quality assessment

All the included studies were prospective or retrospective cohort studies. The quality of the literature was assessed using the NOS scale, which has eight points in three aspects: selectivity, comparability, and outcome. The article scores are listed in Table 1. All articles had NOS score more than five. Therefore, the overall quality of the ten articles was considered good.

### 3.3 Functional outcomes

#### 3.3.1 Wexner score

**FIGURE 2:**
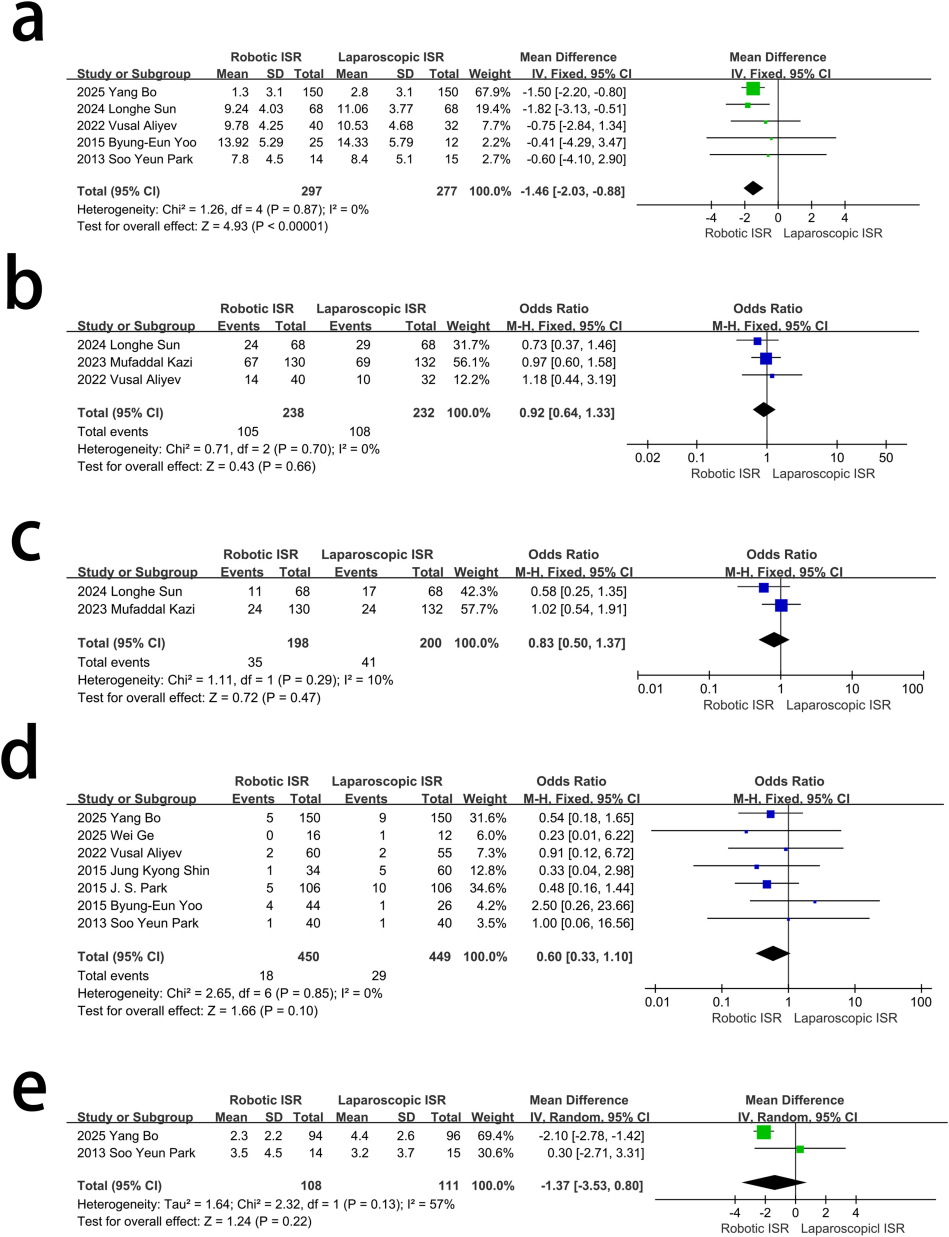
The difference in anus, urinary and sexual functional scores between the two groups. **(a)** Wexner score (negative MD favors robotic). **(b)** Severe Kirwan’s incontinence score, grade ≥ III. **(c)** Major LARS. **(d)** Retention of urine. **(e)** IPSS, International Prostate Symptom Score.

There was no obvious heterogeneity (*I*^2^ = 0%, χ^2^-test *p* = 0.87) in the five studies included. A fixed effect model was adopted, and the results showed that Robotic ISR had a lower Wexner score than Laparoscopic ISR. [MD = –1.46, 95%CI (–2.03 ∼–0.88), *p* < 0.001] (Figure 2a).

#### 3.3.2 Severe Kirwan’s incontinence score

Three studies assessed the difference in severe Kirwan’s incontinence score between the two groups. There was no heterogeneity between the three studies, (*I*^2^ = 0%, χ^2^-test *p* = 0.70) and no statistically significant difference between laparoscopic ISR and robotic ISR in improving the Severe Kirwan’s incontinence score using a fixed effect model.[OR = 0.92, 95%CI (0.64∼1.33), *p* = 0.66] (Figure 2b).

#### 3.3.3 Major LARS

Two studies assessed the difference in Major LARS between the two groups. There were no statistically significant differences between the two groups using a fixed effect model. With mild heterogeneity between the two studies (*I*^2^ = 10%, χ^2^-test *p* = 0.29) [OR = 0.83, 95%CI (0.5∼1.37), *p* = 0.47] (Figure 2c).

#### 3.3.4 Retention of urine

Seven studies assessed the difference in retention of urine between the two groups. There was no obvious heterogeneity among the studies (*I*^2^ = 0%, χ^2^-test *p* = 0.85). Thus, a fixed effect model was used and there was no statistically significant difference between Robotic ISR and the Laparoscopic ISR. [OR = 0.60, 95%CI (0.33∼1.10), *p* = 0.1] (Figure 2d).

#### 3.3.5 IPSS

Two studies assessed the difference in IPSS between the two groups. There was obvious heterogeneity between the two studies (*I*^2^ = 57%, χ^2^-test, *p* = 0.13), and no statistically significant differences between the two groups. [MD = –1.37, 95%CI (–3.53∼0.80), *p* = 0.13] using the random effect modal (Figure 2e).

#### 3.3.6 Male sexual dysfunction

Only two studies evaluated the differences in the occurrence of postoperative male sexual dysfunction. In one study, the mean IIEF-5 score was significantly higher in the robotic ISR group than in the laparoscopic ISR group at 3 and 6 months after surgery (11.6 ± 4.4 in robotic ISR group versus 7.6 ± 2.8 laparoscopic ISR group at 3 months with *p* = 0.006; 13.0 ± 3.9 in robotic ISR group versus 9.0 ± 3.9 laparoscopic ISR group at 6 months with *p* = 0.016) (20). In another study, the incidence of retrograde ejaculation showed a trend in favor of the robotic ISR group but did not reach statistical significance (4 of 33 in robotic ISR group vs. 7 of 35 in laparoscopic ISR group; *p* = 0.124) (16). Overall, robotic surgery demonstrates the potential advantage of preserving male sexual function. However, no data are available for conducting a meta-analysis.

### 3.4 Perioperative outcomes

#### 3.4.1 Complications

**FIGURE 3:**
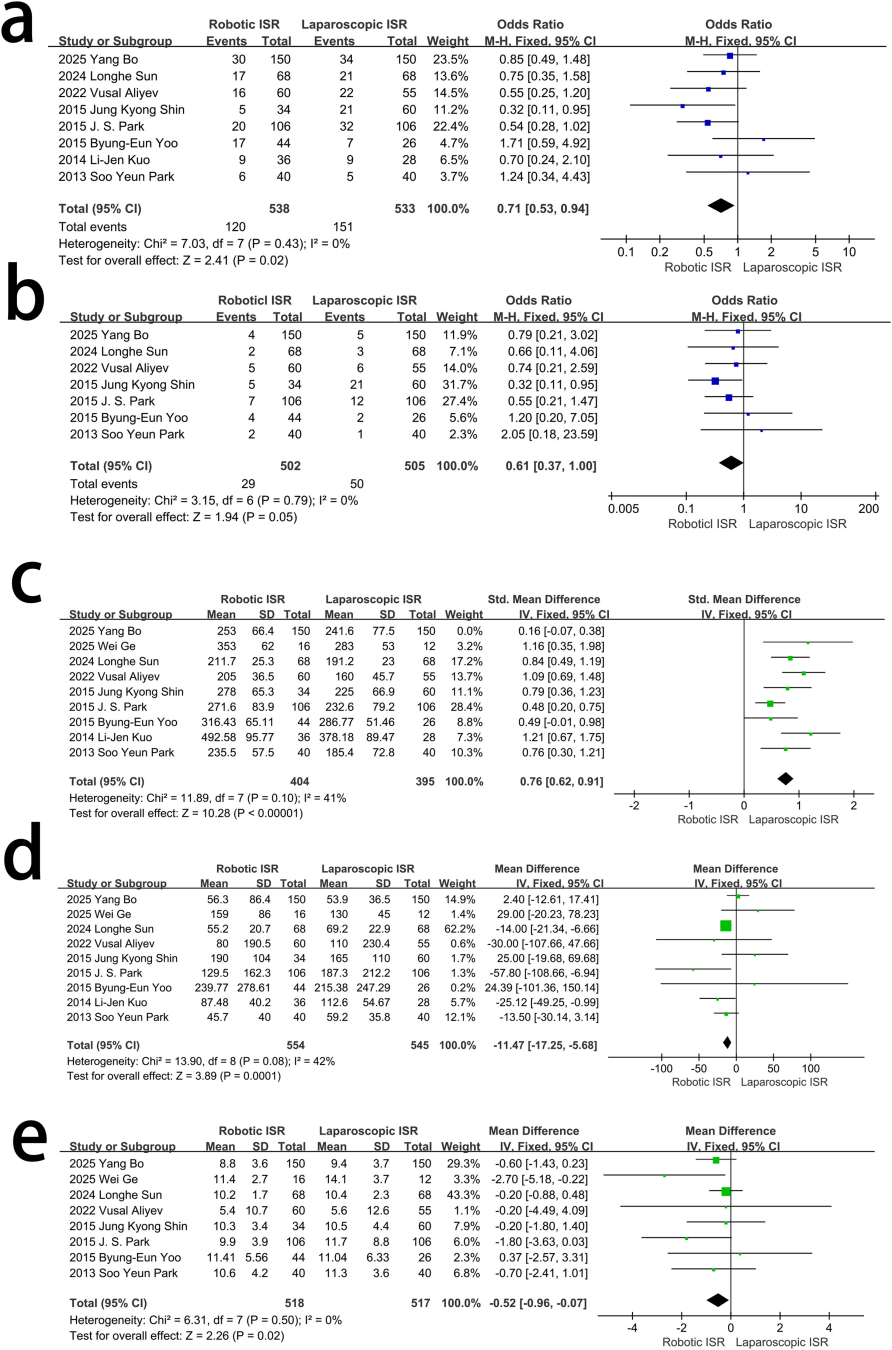
The difference in Perioperative outcomes between the two groups. **(a)** Complications. **(b)** Severe complications, Clavien-Dindo Grade ≥ III. **(c)** Operative time. **(d)** Blood loss in operation. **(e)** Hospitalized time.

Eight studies reported postoperative complications. There was no heterogeneity (*I*^2^ = 0%, χ^2^-test *p* = 0.43). A fixed-effects model was used. The results showed that the overall complications in Robotic ISR was less than that in the Laparoscopic one, and the difference was statistically significant [OR = 0.71, 95% Cl (0.53∼0.94), *p* = 0.02] (Figure 3a).

#### 3.4.2 Severe complications

Seven studies reported severe complications (above grade III in Clavien-Dindo classification), and the heterogeneity was low (*I*^2^ = 0%, χ^2^-test, *p* = 0.79); a fixed-effect model was used. Although there was no significant difference between the two groups, complications appeared to be less severe in the robotic ISR group than in the laparoscopic ISR group [OR = 0.61, 95%CI (0.31–1), *p* = 0.05] (Figure 3b).

#### 3.4.3 Operative time

Nine studies assessed the difference in operative time between the two groups. There was a significant heterogeneity. [*I*^2^ = 74%, χ^2^-test, *p* = 0.0001] and Sensitivity analysis found that the study of Bo et al. **(18)** seems to be the source of heterogeneity, thus it was excluded for the reason of a significant difference in DAV between the two groups in this study and the result indicated that the Robotic ISR will cost more operative time [MD = 0.76, 95%CI (0.62∼0.91), p < 0.001] using Fix effect model with mild heterogeneity [*I*^2^ = 41%, χ^2^-test *p* = 0.10] (Figure 3c).

#### 3.4.4 Blood loss in operation

Nine studies assessed blood loss during surgery with mild heterogeneity [*I*^2^ = 42%, χ^2^-test *p* = 0.08], and a fixed-effect model was adopted. Robotic ISR was associated with less blood loss during surgery than laparoscopic ISR [MD = –11.47, 95%CI-17.25 to–5.68, *p* = 0.0001] using a random-effects model (Figure 3d).

#### 3.4.5 Hospitalized time

Eight studies assessed the difference in hospitalization time between the two groups. No heterogeneity was observed. [*I*^2^ = 0%, χ^2^-test *p* = 5]. Robotic ISR will cost less hospitalization time [MD = –0.52, 95%CI (–0.96∼–0.07), *p* = 0.02] using a fixed effect model (Figure 3e).

There were no significantly different about other perioperative outcomes between the two groups. As shown in Table 3.

**TABLE 3:** Other perioperative outcomes.

Outcomes	No. of studies	No. of patients		MD/OR (95%Cl)	*p*-value	Heterogenetity	
		Robotic	Laparoscopic			*I*^2^ (%)	χ ^2^-test (*p*-value)
Anastomotic leak	9(15–20, 22–24)	648	649	0.93[0.6, 1.45]*	0.74	0	0.76
Anastomotic stenosis	5 (15, 16, 21–23)	286	269	0.85[0.40, 1.81]*	0.66	0	0.65
Fistula	6 (15, 16, 21–24)	320	329	0.48[0.17, 1.36]*	0.17	0	0.83
Abdominal-pelvic infection	8 (15, 16, 19, 20, 22–24)	368	367	0.88[0.34, 2.28]*	0.79	0	0.88
Postoperative bleeding	6(15, 19, 21–24)	258	249	0.70[0.24, 2.07]*	0.52	0	0.68
Intestinal obstruction	8(15, 16, 18, 19, 21–24)	514	590	0.72[0.37, 1.40]*	0.34	0	0.78
Diet	6(16, 19–22, 24)	276	272	–0.04[0.32, 0.24]#	0.8	47	0.09
First flatus	4 (15, 20, 22, 23)	184	175	–0.12[–0.33, 0.09]#	0.26	49	0.12

*Odds ratio (OR) for dichotomous; #: mean differences (MD) for continuous.

### 3.5 Oncology outcomes

**FIGURE 4:**
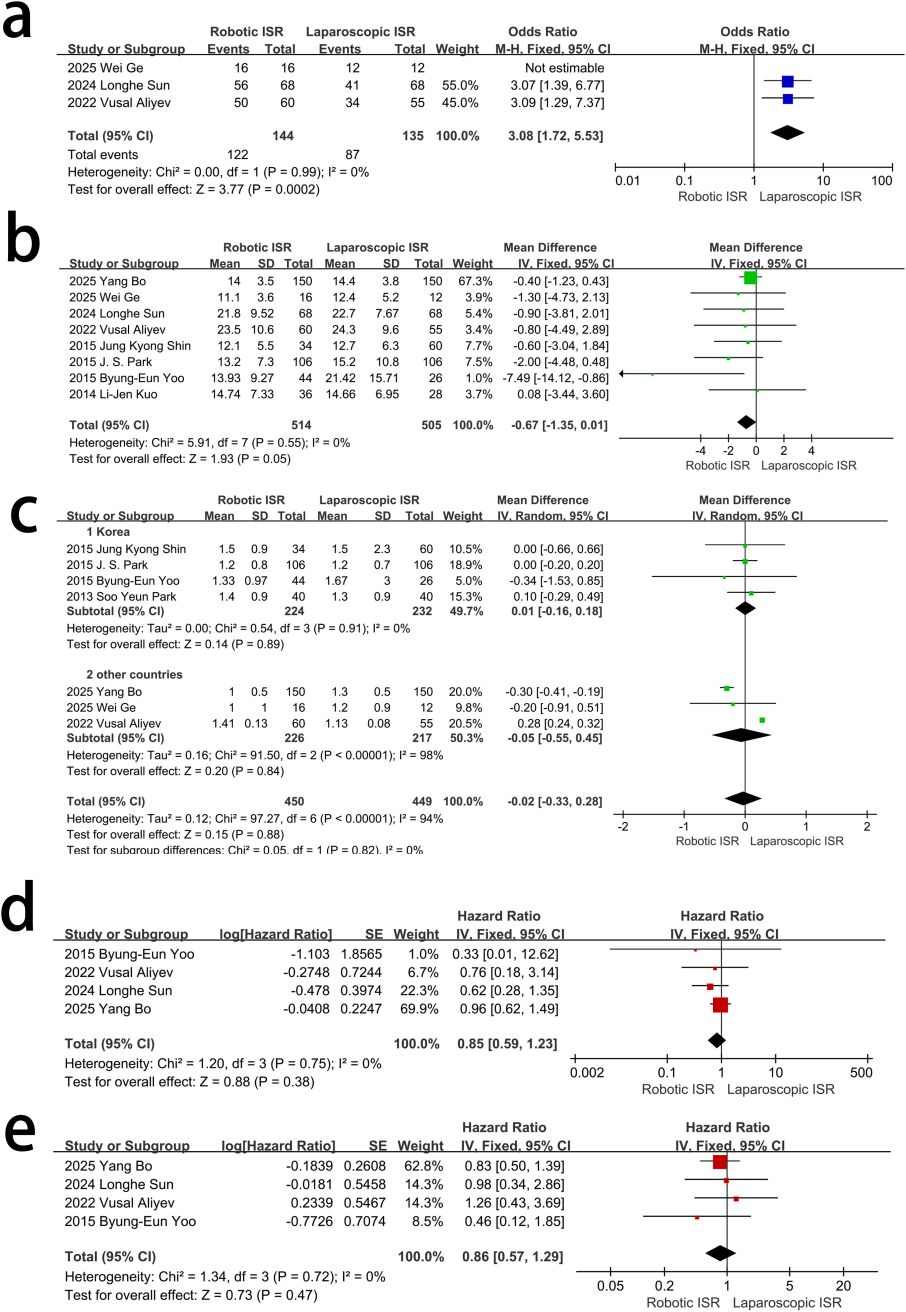
The difference in oncology outcomes between the two groups. **(a)** Integrity of mesorecta. **(b)** Retrieved Lymph. **(c)** Distal resection margin. **(d)** Overall survival. **(e)** Diseases free survival.

Robotic ISR had better integrity of mesorecta than laparoscopic ISR [OR = 3.08, 95%CI (1.72∼5.53), *p* = 0.0002]. No obvious heterogeneity was observed among the three studies. [*I*^2^ = %, χ^2^-test *p* = 0.99] (Figure 4a). Other short- and long-term oncological outcomes did not differ significantly between the two groups (Figures 4b–e). Regarding the distal resection margin, the seven studies had obvious heterogeneity (*I*^2^ = 94%, χ^2^-test *p* < 0.0001) [MD = –0.02, 95%CI (–0.33∼0.28), *p* = 88]. Subgroup analysis indicated that there was no heterogeneity among all Korean studies (*I*^2^ = 0%, χ^2^-test *p* = 0.89), and there was still no significant difference within this subgroup. [MD = 0.01, 95%CI (–0.16∼0.18), *p* = 0.91] (Figure 4c).

### 3.6 Publication bias evaluation

**FIGURE 5:**
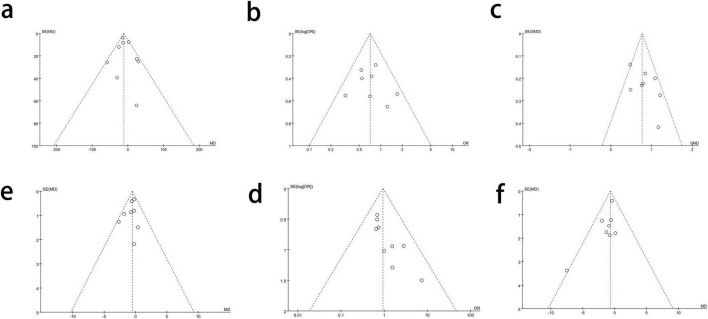
Funnel plots to test the publication bias. **(a)** Blood loss. **(b)** Complication. **(c)** Operation time. **(d)** Hospitalized time. **(e)** Anastomotic leak. **(f)** Retrieved lymph nodes.

Funnel plots were drawn for the projects that included more than eight articles and had very low heterogeneity to test for publication bias. Most of the scatter plots were symmetrically distributed, indicating an acceptable publication bias in this meta-analysis (Figure 5).

## 4 Discussion

Intersphincteric resection significantly increases the rate of anal preservation in patients with low rectal cancer. The major challenge in ISR is improving the postoperative anal function in patients. Poor anal function after ISR may be due to the following reasons: (1) the removal of the rectal ampulla results in the loss of storage function of the new rectum, which is an inevitable problem in all radical rectal cancer surgeries. (2) The removal of part or all of the internal sphincter, which damages the anal structure, is an unavoidable problem in ISR. (3) Separation of the intersphincteric groove may damage autonomic nerves, intersphincteric groove receptors, and rectal longitudinal muscle fibers. (4) Complications related to surgery may lead to chronic inflammatory responses or scarring in the pelvic area. Therefore, enhancing the quality of surgery and reducing secondary injuries and surgical complications are key to protecting anal function. In laparoscopic ISR surgeries, surgeons often encounter problems such as a limited field of vision and narrow operating space, which make ISR extremely difficult, especially for obese and male patients. **(25, 26)**. Robotic ISR seems to overcome these problems, thereby improving the quality of surgery and enhancing anal function.

Our meta-analysis revealed that robotic ISR has lower Wexner scores and a lower incidence of complications than Laparoscopic ISR for the first time. Two other meta-analyses suggested that robotic ISR had similar postoperative complications and oncological outcomes as Laparoscopic ISR and a lower conversion rate, less blood loss, and longer operation time (27, 28). However, these two studies included only five studies, with a total sample size of only 510, no publication bias analysis, and very little evaluation of functional results. What’s more, they did not pool Wexner or LARS scores quantitatively. Our study adds to the latest studies conducted in recent years. However, their conclusion remains controversial. Bo et al. reported significantly better LARS and IPSS scores with robotics, whereas Kazi et al. found no difference in incontinence or LARS between groups. Therefore, our study incorporated these new documents and conducted an integrated analysis of organ function, pre-operative conditions and oncological outcomes.

Functional outcomes were the primary outcomes of our study, and we evaluated anal, urinary, and male sexual function. The methods for assessing anal function after sphincter preservation surgery usually involve questionnaires that can truly reflect the patient’s quality of life, including the Wexner score (29), Kirwan’s incontinence score (30), Specific LARS Assessment Scale (31, 32), POLARS score (33), and Stool Diary (34). However, there are differences and deficiencies in various incontinence scoring systems, and there is still a lack of a comprehensive scoring system (31, 35); therefore, a combination of multiple scales is necessary to conduct a more comprehensive evaluation of fecal incontinence and the specific symptoms of LARS. Our research discovered that robotic ISR has a potential advantage over laparoscopic ISR in preserving anal function for the first time, and the Wexner score of the robotic ISR group was lower in a meta-analysis that included five studies (15, 18–20, 23). Robotic ISR did not demonstrate statistically significant advantages in severe Kirwan score grades or major LARS. This may be because only three (15, 17, 23) and two (15, 17) studies were included in the corresponding meta-analyses, respectively, and different results may have been obtained if the sample size had been larger. A similar situation occurred in the evaluation of the IPSS and sexual function. From another perspective, there was no significant difference between the two groups in terms of LARS and the incontinence group. This might suggest that the benefits obtained by the robot group in the Weisner score could be relatively limited, or it could depend on the specific circumstances. Among the studies included in our research, the authors also used several other indicators to evaluate organ function, including stool frequency (23), frequency of bowel movements (18), Kelly scoring criteria (22), and International Index of Erectile Function (20); however, a meta-analysis cannot be carried out due to a lack of data. J. Park reported little difference in intestinal function between the two groups after 24 months but did not specify the evaluation method (16).

This potential advantage of robotic ISR in anus function may be due to the higher precision of robotic surgery, which can reduce injuries to the pelvic floor fascia, nerves, and external sphincter muscles. Robotic surgery would result in greater integrity of the mesorectum in the procedure of TME, which is anatomical evidence (Figure 4a). However, we have not found imaging studies that would strengthen this argument. By detecting the differences in the electrophysiological activities of pelvic nerves in the two groups, it may be possible to provide relevant evidence, which may potentially be a direction for future research.

Postoperative complications may also weaken anal function. Therefore, we paid special attention to postoperative complications. The mechanism of functional impairment after ISR differs from that observed after a simple low anterior resection. Due to a lower anastomosis site, internal sphincter resection, accidental injury to the pelvic nerves and external sphincter, and a higher incidence of postoperative complications, LARS symptoms after ISR are more severe, last longer, and manifest more often as fecal incontinence (36, 37). Unlike previous meta-analyses (27, 28), we found that the overall number of Robotic ISR procedures was fewer than that of laparoscopic surgery, and severe complications, which might be due to the increase in the sample size. A similar tendency was observed for severe complications. Therefore, the improvement in anal function in the robotic ISR group may be attributed to the reduction in the incidence of complications. However, no statistically significant differences were observed between the two groups in terms of anastomotic leakage and anastomotic stenosis. This may require further expansion of the sample size. In addition, Robotic ISR takes more time with less operative bleeding than laparoscopic ISR. The more detailed it is, the more time it requires to complete. This finding is consistent with the characteristics of robotic surgeries. However, the extension of the operation time did not lead to a delay in postoperative recovery, which was reflected in the hospitalization time, postoperative diet, and flatus (Figures 3c–e). These are consistent with those of a previous meta-analysis (27, 38).

In oncology, laparoscopic surgery is associated with a high rate of incomplete mesorectal excision (39). Our meta-analysis revealed that robotic ISR was better at preserving the complete rectal mesentery. However, there was no significant difference between the two groups in terms of lymph node retrieval or distal resection margins. These results indicate that the advantage of robotic surgery lies in its ability to achieve a more precise membrane anatomical level than laparoscopic surgery. However, the advantages of robotic ISR at the pathological and anatomical levels are not reflected in long-term survival. Although the sample size was larger, our study did not find any statistically significant differences in OS and DFS between the two groups, which is consistent with previous meta-analyses (28). The difference is that we used HR as the evaluation indicator, whereas previous studies used the 3-year survival rate. (28) Due to data limitations, we were unable to conduct a meta-analysis of the local recurrence-free survival rates. A recently published multicenter randomized controlled trial indicated that robotic surgery can reduce the local recurrence rate of rectal cancer compared with laparoscopic surgery (40). This study is worthy of reference, although a subgroup analysis was not conducted for ISR surgery. However, it can be used as potential data for mining.

The advantages of robotic surgery enable its indications to be expanded beyond ISR and surgeries in other narrow spaces, such as surgeries for tumors at the gastroesophageal junction. Although robotic ISR has certain advantages over laparoscopic ISR in terms of safety and anal function, it still has some disadvantages. For real-world adoption, cost is crucial. Robotic surgery is generally perceived as being more expensive than laparoscopic surgery (41, 42). Among the studies included in this systematic review, only one mentioned cost, and the results were the same (16). However, we should not only focus on the cost but also consider the cost-effectiveness. Furthermore, indirect costs incurred due to losses such as lost wages, productivity, and costs resulting from the need for home care and others were not mentioned in most studies. It is also necessary to note that, for a long time, the robotic surgery market has been dominated by a single major supplier. With the emergence of new competitors, costs should be reduced. Additionally, the development of medical insurance may gradually solve these problems. In addition, the lack of tactile feedback is another disadvantage of robotic surgery.

Our study also has some other limitations: (1) Some studies have shown that tumor location, ISR type, neoadjuvant therapy, and anastomotic leakage are independent risk factors for major LARS after minimally invasive sphincter-sparing surgery for low rectal cancer (43). Among the studies included in this systematic review, these variables showed significant differences between the two groups. In several studies, the tumor distance from the anus verge in the robotic ISR group was closer (15, 18, 19), and there was a significant difference in the proportion of neoadjuvant therapy between the two groups (19, 20) Many studies did not provide information on the types of ISR (15, 16, 19–21, 24). These may be confounding factors affecting the stability of the meta-analysis or may be the cause of heterogeneity. We attempted to conduct stratified or subgroup analyses using DAV and ISR types in the meta-analysis but failed because of the limited number of studies and insufficient data. (2) The measurement time for anal function was not uniform, varying from half a year to two years. Conducting a meta-analysis of the measurement data from these studies may affect result stability. However, because the changes in anal function after half a year to two years post-operation are relatively small (18, 44), and the recovery of anal function after surgery always tends to be better, this approach, although not perfect, is reasonable. (3) Another primary limitation of this meta-analysis is the absence of included randomized controlled trials (RCTs). This implies that baseline differences between the intervention and control groups could significantly influence the observed outcomes. This fundamental limitation reduces the strength of the conclusions that can be drawn regarding the efficacy of the intervention and underscores the need for future high-quality RCTs to provide more definitive evidence on this topic. (4) None of the studies originated from North America or Western Europe. Although all cases originated from Asia, the ethnicities were not the same. (5) Only English literature was included and functional results of some studies could not be obtained, which may indicate publication bias. There are still relatively few literatures evaluating organ functions, and it is difficult to control for their heterogeneity. This might limit the stability of the conclusion. (6) Some studies did not provide data on preoperative anal function. Postoperative rehabilitation exercises may also affect anal function (44–46), but the included literature did not describe this in detail.

## 5 Future research directions

Based on these limitations, we suggest that future research should focus on the following points. First, the assessment indicators for anal function should be diversified, including physiological parameters [mean resting pressure and maximal squeezing pressure, etc. (3, 47)], assessment of anal function [fecal incontinence severity index, etc. (48)], and assessment of quality of life [e.g., the New Cleveland Clinic Colorectal Cancer Quality of Life Questionnaire (49)]. Second, the measurement of functional data should cover multiple time points within at least two years after surgery, as the function of the anus after surgery will naturally change over time (44, 50). Third, subgroup or stratified analysis should be fully conducted, including but not limited to DAV, ISR type, ISR route (total transabdominal or combined), coloanal anastomosis, radiation therapy intensity, postoperative rehabilitation exercises, and treatment methods.

## 6 Conclusion

Robotic ISR has a potential advantage over Laparoscopic ISR in preserving better anal function, which may be due to the fact that Robotic ISR can achieve better surgical anatomy and reduce possible side injuries and complications of surgery. Due to the heterogeneity of the study and the limitation of the sample size, this result needs to be viewed with caution, and multicenter RCTs with harmonized functional assessments and DAV-adjusted or ISR type-adjusted analyses are needed.

## Data Availability

The original contributions presented in the study are included in the article/Supplementary material, further inquiries can be directed to the corresponding authors.
