# Supplementary material for: Does robotic intersphincteric resection better preserve anal function in low rectal cancer: a systematic review and meta-analysis?
Source: Front Med (Lausanne). 2026 May 21;13:1778921. doi: 10.3389/fmed.2026.1778921 (PMC13233208; doi:10.3389/fmed.2026.1778921)
Supplement: Supplementary file 1 [file Data_Sheet_1.pdf]

search strategy

#1Rectal Neoplasms[Mesh]

#2 Neoplasms, Rectal[Title/Abstract]

#3 Rectal Neoplasm[Title/Abstract]

#4 Neoplasm, Rectal[Title/Abstract]

#5 Rectum Neoplasms[Title/Abstract]

6 Neoplasm, Rectum[Title/Abstract]

#7 Rectum Neoplasm[Title/Abstract]

#8 Rectal Tumors[Title/Abstract]

#9 Rectal Tumor[Title/Abstract]

#10 Tumor, Rectal[Title/Abstract]

#11 Cancer of Rectum[Title/Abstract]

#12 Rectum Cancers[Title/Abstract]

#13 Cancer of the Rectum[Title/Abstract]

#14 Rectal Cancer[Title/Abstract]

#15 Cancer, Rectal[Title/Abstract]

#16 Rectal Cancers[Title/Abstract]

#17 Rectum Cancer[Title/Abstract]

#18 Cancer, Rectum[Title/Abstract]

#19= #1 OR #2 OR #3 OR #4 OR #5 OR #6 OR #7 OR #8 OR #9 OR #10 OR #11 OR #12 OR #13 OR #14 OR #15 OR #16 OR #17 OR #18

#20 Robotic Surgical Procedures[Mesh]

#21 Procedure, Robotic Surgical[Title/Abstract]

#22 Procedures, Robotic Surgical[Title/Abstract]

#23 Robotic Surgical Procedure[Title/Abstract]

#24 Surgical Procedure, Robotic[Title/Abstract]

#25 Robotic-Assisted Surgery[Title/Abstract]

#26 Robotic-Assisted Surgeries[Title/Abstract]

#27 Robotic Assisted Surgery[Title/Abstract]

#28 Surgery, Robotic-Assisted[Title/Abstract]

#29 Surgical Procedures, Robotic[Title/Abstract]

#30 Robot-Assisted Surgery[Title/Abstract]

#31 Robot-Assisted Surgeries[Title/Abstract]

#32 Robot Assisted Surgery[Title/Abstract]

#33 Surgery, Robot-Assisted[Title/Abstract]

#34 Robot Surgery[Title/Abstract]

#35 Robot Surgeries[Title/Abstract]

#36 Surgery, Robot[Title/Abstract]

#37 Robot-Enhanced Procedures[Title/Abstract]

#38 Procedure, Robot-Enhanced[Title/Abstract]

#39 Robot-Enhanced Procedure[Title/Abstract]

#40 Robot Enhanced Procedures[Title/Abstract]

#41 Robot-Enhanced Surgery[Title/Abstract]

#42 Robot-Enhanced Surgeries[Title/Abstract]

#43 Robot Enhanced Surgery[Title/Abstract]

#44 Surgery, Robot-Enhanced[Title/Abstract]

#45 robotic[Title/Abstract];

#46 robot[Title/Abstract];

#47da Vinci[Title/Abstract]

#48=#20 OR #21 OR #22 OR #23 OR #24 OR #25 OR #26 OR #27 OR #28 OR #29 OR #30 OR #31 OR #32 OR #33 OR #34 OR #35 OR #36 OR #37OR #38 OR #39 OR #40 OR #41 OR #42 OR #43 OR #44 OR #45 OR #46 OR #47

#49 intersphincteric[Title/Abstract]

#50 ISR[Title/Abstract]

#51 sphincterectomy[Title/Abstract]

#52=#49 OR #50 OR #51

search strategy： #19 AND #48 AND #52
